# Effect of Acute Negative and Positive Energy Balance on Basal Very-Low Density Lipoprotein Triglyceride Metabolism in Women

**DOI:** 10.1371/journal.pone.0060251

**Published:** 2013-03-22

**Authors:** Elena Bellou, Maria Maraki, Faidon Magkos, Helena Botonaki, Demosthenes B. Panagiotakos, Stavros A. Kavouras, Labros S. Sidossis

**Affiliations:** 1 Department of Nutrition and Dietetics, Harokopio University, Athens, Greece; 2 Department of Internal Medicine, Center for Human Nutrition and Atkins Center of Excellence in Obesity Medicine, Washington University School of Medicine, St. Louis, Missouri, United States of America; 3 Department of Health, Human Performance and Recreation, University of Arkansas, Fayetteville, Arkansas, United States of America; 4 Department of Internal Medicine, Sealy Center on Aging, Institute for Translational Sciences and Shriners Hospital for Children, University of Texas Medical Branch at Galveston, Galveston, Texas, United States of America; Scientific Directorate, Bambino Hospital, Italy

## Abstract

**Background:**

Acute reduction in dietary energy intake reduces very low-density lipoprotein triglyceride (VLDL-TG) concentration. Although chronic dietary energy surplus and obesity are associated with hypertriglyceridemia, the effect of acute overfeeding on VLDL-TG metabolism is not known.

**Objective:**

The aim of the present study was to investigate the effects of acute negative and positive energy balance on VLDL-TG metabolism in healthy women.

**Design:**

Ten healthy women (age: 22.0±2.9 years, BMI: 21.2±1.3 kg/m^2^) underwent a stable isotopically labeled tracer infusion study to determine basal VLDL-TG kinetics after performing, in random order, three experimental trials on the previous day: i) isocaloric feeding (control) ii) hypocaloric feeding with a dietary energy restriction of 2.89±0.42 MJ and iii) hypercaloric feeding with a dietary energy surplus of 2.91±0.32 MJ. The three diets had the same macronutrient composition.

**Results:**

Fasting plasma VLDL-TG concentrations decreased by ∼26% after hypocaloric feeding relative to the control trial (*P* = 0.037), owing to decreased hepatic VLDL-TG secretion rate (by 21%, *P* = 0.023) and increased VLDL-TG plasma clearance rate (by ∼12%, *P* = 0.016). Hypercaloric feeding increased plasma glucose concentration (*P* = 0.042) but had no effect on VLDL-TG concentration and kinetics compared to the control trial.

**Conclusion:**

Acute dietary energy deficit (∼3MJ) leads to hypotriglyceridemia via a combination of decreased hepatic VLDL-TG secretion and increased VLDL-TG clearance. On the other hand, acute dietary energy surplus (∼3MJ) does not affect basal VLDL-TG metabolism but disrupts glucose homeostasis in healthy women.

## Introduction

Elevated plasma triglyceride (TG) concentration is an independent risk factor for coronary heart disease, especially in women [1]. Long-term energy imbalance can lead either to weight gain, associated with an increase in plasma TG concentrations [2], or weight loss, associated with a decrease in plasma TG concentrations [3]. However little is known about the acute effect of positive or negative energy balance, independently of changes in body weight and composition, on TG metabolism.

Very low density lipoprotein (VLDL) is the major carrier of TG in plasma during postabsorptive conditions [4]. It has been shown previously that long term dietary energy restriction leading to weight loss decreases fasting total TG and VLDL-TG concentrations after a period of weight stabilization, and this effect is mediated by a reduction in VLDL-TG secretion rate from the liver without changes in VLDL-TG plasma clearance rate [3], [5]. Recent data indicate that decreasing dietary energy intake for a short period of time (5 days; energy deficit of 3.8 MJ per day) can decrease fasting plasma TG concentrations [6], and we have shown previously that even a single day of dietary energy restriction (energy deficit of 2 MJ) mildly reduces fasting and postprandial plasma TG concentrations [7]. However, in a subsequent study, we failed to observe any changes in basal VLDL-TG kinetics, which may have been due to the energy deficit (2 MJ) being inadequate in these subjects [8].

On the other hand, positive energy balance induced by increasing dietary energy intake also affects plasma TG homeostasis independently of weight gain [9], [10], [11], [12]. However the effect of hypercaloric feeding on TG concentrations appears to depend on the macronutrient that is ingested in excess: carbohydrate and particularly fructose overfeeding for a short period of time (4–6 days) increases total plasma TG and VLDL-TG concentrations [11], [13], [14], whereas fat overfeeding for a few days decreases total plasma TG and VLDL-TG concentrations [9], [10], [15]. The mechanisms responsible for these acute effects of overfeeding remain elusive, however, under conditions of isocaloric feeding, both an increase in hepatic VLDL-TG secretion rate [16] and a decrease in VLDL-TG plasma clearance rate [17] have been suggested to be responsible for the increase in plasma TG concentration after short-term (2–5 weeks) high-carbohydrate diets relative to diets with greater fat content. Nevertheless, the effect of hypercaloric feeding with a mixed diet on VLDL-TG metabolism is not known.

Therefore, the aim of the present study was to assess the acute effects of negative and positive energy balance on VLDL-TG metabolism in healthy women.

## Subjects and Methods

### Subjects

Ten healthy, lean, sedentary women volunteered for the study (Table 1). Exclusion criteria included irregular menses, amenorrhea, polycystic ovary syndrome, pregnancy, acute or chronic illness, use of medications (including oral contraceptives) or dietary supplements, smoking, regular alcohol consumption (>1drink per day), regular exercise participation (>1 time per week), and being on a special diet or having experienced weight fluctuations ≥2 kg at any time during the last 6 months. The Ethics Committee of Harokopio University approved the study protocol and all subjects gave written informed consent.

**Table 1:** Baseline characteristics of subjects (n = 10).

	Mean±SD
Age (yrs)	22.0±2.9
Weight (kg)	58.9±6.9
Height (m)	1.67±0.07
Body mass index (kg/m^2^)	21.2±1.3
Lean body mass (kg)	38.0±3.7
Body fat mass (kg)	18.5±4.4
Body fat (%)	31.2±4.5
Resting energy expenditure (MJ)	5.0±0.6

### Preliminary testing

All preliminary tests were carried out during screening, approximately 1–2 weeks before the beginning of the experiment. Weight and height were measured and an overnight fasting blood sample was drawn for hematological and biochemical evaluations. Subjects were healthy on the basis of medical examination and routine laboratory tests; all were normoglycemic and normolipidemic. Total body fat mass and fat-free mass were determined with dual energy x-ray absorptiometry (model DPX-MD; Lunar, Madison,WI). Resting energy expenditure (REE) was measured by indirect calorimetry (Vmax229D; Sensormedics, Yorba Linda, CA) in the morning, after subjects remained rested for at least 30 min [18].

### Experimental protocol

We used a paired cross-over design, in which all subjects were evaluated after three one-day trials (isocaloric diet, hypocaloric diet, and hypercaloric diet) in random order and at least one week apart. The phase of the menstrual cycle was not taken into account in scheduling the studies because we have previously shown that VLDL-TG kinetics are not affected by menstrual cycle phase [19]. A stable isotopically labeled tracer infusion was performed on the day after each trial, following an overnight fast. Subjects were instructed to keep a record of their diet and to refrain from exercise for 2 days before each trial, and to avoid alcohol and caffeine consumption for 1 day before each trial. They were also instructed to replicate their diet on the day preceding the first trial during the subsequent trials (i.e. purchase the same type and brand of food, use the same cooking methods and portions, etc.), in order to ensure the same pre-trial energy and macronutrient intakes. Food records were analyzed by using Diet Analysis Plus 8 (Cengage Learning, Florence, KY).

#### Isocaloric diet

Subjects were instructed to follow a prescribed isocaloric diet which provided their estimated daily energy needs for weight maintenance, calculated by multiplying the measured REE with an activity factor of 1.4 representative of their very light to light physical activity habits [20]. Subjects were thus assumed to be on zero energy balance during the control trial.

#### Hypocaloric diet

Subjects were instructed to follow a prescribed hypocaloric diet, which provided their estimated daily energy needs for weight maintenance minus 3 MJ.

#### Hypercaloric diet

Subjects were instructed to follow a prescribed hypercaloric diet, which provided their estimated daily energy needs for weight maintenance plus 3 MJ. In order to achieve maximum adherence to the prescribed diet, the energy surplus was provided with the use of two high-caloric (6 KJ/ml), fiber-free energy drinks (200 ml/drink; 50% of energy from carbohydrate, 15% from protein and 35% from fat; Fresubin, Fresenius Kabi).

All diets were designed to provide 50% of energy from carbohydrate, 20% from protein and 30% from fat. The macronutrient content of the experimental diets is shown in Table 2. For each trial subjects were asked to abstain from exercise and carry out only the activities of daily living.

**Table 2:** Dietary energy intake and macronutrient content of the experimental diets.

	Isocaloric Diet	Hypocaloric Diet	Hypercaloric Diet
Dietary energy intake (MJ)	6.79±1.30	3.91±1.10*^†^	9.70±1.34*
Energy balance (MJ)	0	−2.89±0.42*^†^	2.91±0.32*
Carbohydrate (g)	216±34	127±34*^†^	307±33*
Protein (g)	80±18	47±15*^†^	100±35*
Fat (g)	52±18	31±15*^†^	73±18*

Values are means ± SD (n = 10). ^*^ Significantly different from isocaloric diet, P<0.05. ^†^ Significantly different from hypercaloric diet, P<0.05.

### Tracer infusion study

The morning following each of the three experimental trials, subjects arrived at the laboratory at approximately 0800 h, after an overnight fast. A catheter was inserted in a forearm vein to administer stable isotopically labeled tracers and a second catheter was inserted in a contralateral hand vein for blood sampling, which was kept warm with a heating pad until the end of the metabolic study. Subjects were given 1 h to relax and familiarize with the catheters. During this time, a 24-hour diet recall was taken to examine their adherence to the prescribed diet on the previous day. At 0900 h, a baseline blood sample was obtained and immediately after a bolus of [1,1,2,3,3-^2^H_5_]glycerol (75 µmol/kg body weight; Goss Scientific Instruments, Essex, UK), dissolved in normal saline, was administered through the catheter in the forearm vein. Blood samples were obtained at 15 min and then every hour after tracer administration for a total of 6 hours. Catheters were flushed with saline every 30 min to maintain patency. Subjects remained fasted (except for water) in a sitting position until the end of the metabolic study.

### Sample collection, processing, and analysis

Blood samples were collected in precooled potassium-EDTA Monovettes (Sarstedt, Leicester, UK), and immediately placed on ice. Plasma was separated by centrifugation within 30 minutes of collection. A 3-ml aliquot of plasma was transferred into plastic culture tubes and kept in the refrigerator for immediate isolation of VLDL, and the remaining plasma samples were stored at −80°C until analyses. The VLDL fraction was prepared by density-gradient ultracentrifugation, VLDL-TG were isolated by thin-layer chromatography, hydrolyzed, and VLDL-TG-bound glycerol was derivatized with heptafluorobutyric anhydride, as previously described [21], [22]. The tracer to tracee ratio (TTR) of glycerol in VLDL-TG was measured by gas chromatography-mass spectrometry (MSD 5973 system; Hewlett-Packard, Palo Alto, CA) by selectively monitoring the ions at mass-to-charge ratios 467 and 472 [21], [22].

Determination of plasma glucose, total plasma TG and VLDL-TG concentrations was performed by enzymatic colorimetric methods using commercially available kits (Alfa Wassermann Diagnostics, Woerden, The Netherlands) on an automated analyzer (ACE Schiapparelli Biosystems, Fairfield, NI). A separate blood sample was collected into non-heparinized serum tubes (Sarstedt, Leicester, UK), allowed to clot, spun in a centrifuge and then aliquoted and frozen immediately at −80°C, until measurement of insulin with a commercially available immunoenzymetric fluorescent method (ST AIA-PACK IRI, Tosoh Medics, San Francisco, CA) on an automated analyzer (Tosoh AIA 600II, Tosoh Medics, Inc., San Francisco, CA). All samples from each subject's trials were analyzed in the same batch.

### Calculations

The fractional turnover rate (FTR, pools·h^−1^) of VLDL-TG was determined by monoexponential analysis of VLDL-TG-glycerol TTR data [23], [24]. The hepatic secretion rate of VLDL-TG ( µmol·min^-1^) was calculated as FTR × C × PV/60, where C is the concentration of VLDL-TG in plasma and PV is the plasma volume (55 ml per kg of fat-free mass [25]). It was assumed that VLDL-TG volume of distribution equals PV because VLDL particles are restricted to the plasma compartment [26]. The plasma clearance rate of VLDL-TG (ml·min^-1^), which is an index of the efficiency of VLDL-TG removal from the circulation via all possible routes, was calculated by dividing the rate of VLDL-TG disappearance (which equals the secretion rate at steady state) by the plasma concentration of VLDL-TG. The mean residence time (MRT, min) of VLDL-TG in the circulation was calculated as 60/FTR. Insulin resistance was assessed by HOMA index as follows: fasting serum insulin ( µU/mL) × fasting plasma glucose (mmol/L)/22.5 [27].

### Statistical Analysis

All datasets were tested for normality by using the Kolmogorov-Smirnov criterion. Normally distributed variables are presented as mean±SD, whereas non-normally distributed variables were log-transformed for analyses and back-transformed for presentation as means with 95% confidence intervals. Generalized estimating equations (GEE) were fitted to evaluate differences among the three experimental trials (encoded as dummy variables). For all the dependent variables, the normal distribution was used for fitting GEE, with the identity as the link function. The unstructured formation of the correlation matrix was used after comparing various scenarios using the corresponding QIC (Quasi likelihood under the Independence criterion for model's goodness-of-fit). Post-hoc analysis for comparing mean values among trials was applied by using the Bonferroni correction rule to adjust for the inflation of type-I error due to multiple comparisons. All statistical analyses were carried out with SPSS 19 for Windows (IBM SPSS, Chicago, IL).

## Results

### Dietary energy intake and nutrient content of experimental diets

There were no significant differences among trials in energy and macronutrient intakes during the two days preceding the experimental feeding day (all *P*-values >0.05). Compared with the control condition (isocaloric diet; zero energy balance), subjects were in a negative energy balance of ∼3 MJ during the hypocaloric diet and a positive energy balance of ∼3 MJ during the hypercaloric diet (Table 2).

### Metabolic profile

Fasting plasma glucose concentration was significantly greater after hypercaloric than after isocaloric (P = 0.042) or hypocaloric (P = 0.001) feeding; serum insulin concentration was also greater after hypercaloric compared to hypocaloric feeding (P<0.001) (Table 3). Total plasma TG and VLDL-TG concentrations were significantly lower after hypocaloric compared to isocaloric (P<0.05) and hypercaloric (P<0.01) feeding, but not different between the hypercaloric and isocaloric trials (P = 1.000) (Table 3).

**Table 3:** Effects of the experimental diets on basal metabolic profile.

	Isocaloric Diet	Hypocaloric Diet	Hypercaloric Diet
Glucose (mmol·L^−1^)	5.32±0.16	5.29±0.32^†^	5.50±0.28*
Insulin (pmol·L^−1^)	5.40 (4.19, 6.95)	4.41 (3.03, 6.42) ^†^	6.27 (4.67, 8.40)
HOMA-IR index	1.28 (0.98, 1.67)	1.03 (0.69, 1.54) ^†^	1.53 (1.11, 2.10)
Total triglyceride (mmol·L^−1^)	0.73±0.18	0.63±0.19*^†^	0.73±0.27
VLDL-triglyceride (mmol·L^−1^)	0.35±0.13	0.26±0.14*^†^	0.39±0.22

Values are means ± SD or means with 95% CI (n = 10). *Significantly different from isocaloric diet, P<0.05. ^†^ Significantly different from hypercaloric diet, P<0.01. Abbreviations: HOMA-IR, Homeostasis Model of Assessment - Insulin Resistance; VLDL, very low density lipoprotein.

### VLDL-TG kinetics

**Figure 1:**
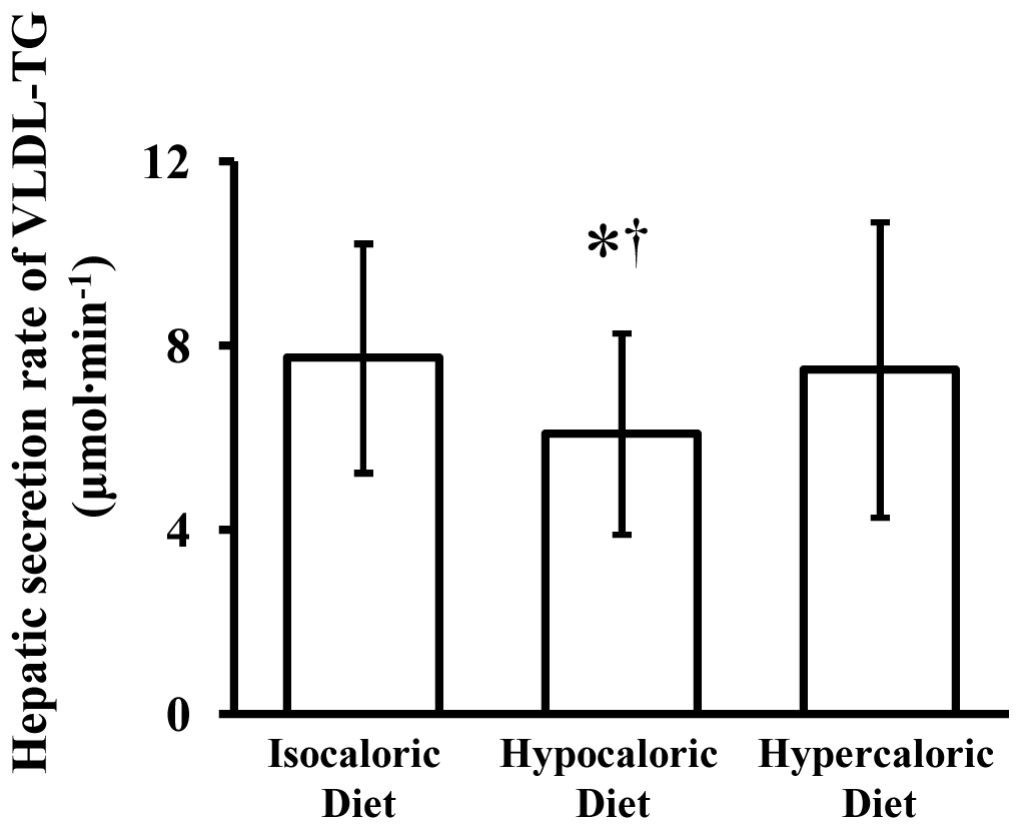
Effects of hypocaloric or hypercaloric feeding relative to isocaloric feeding on the hepatic secretion rate of very low density lipoprotein triglyceride (VLDL-TG). Data are means ± SD (n = 10). *Significantly different from isocaloric diet, P = 0.023.^†^ Significantly different from hypercaloric diet, P = 0.036.

**Figure 2:**
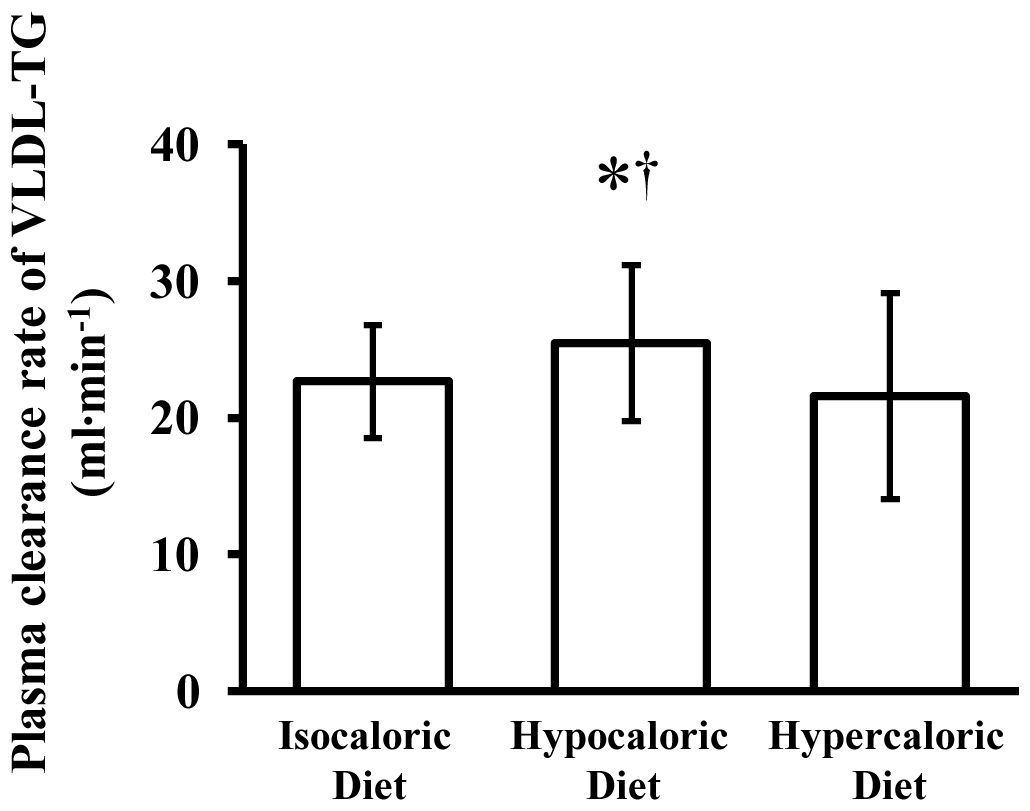
Effectsof hypocaloric or hypercaloric feeding relative to isocaloric feeding on the plasma clearance rate of very low density lipoprotein triglyceride (VLDL-TG). Data are means ± SD (n = 10). *Significantly different from isocaloric diet, P = 0.016.^†^ Significantly different from hypercaloric diet, P<0.001.

**Figure 3:**
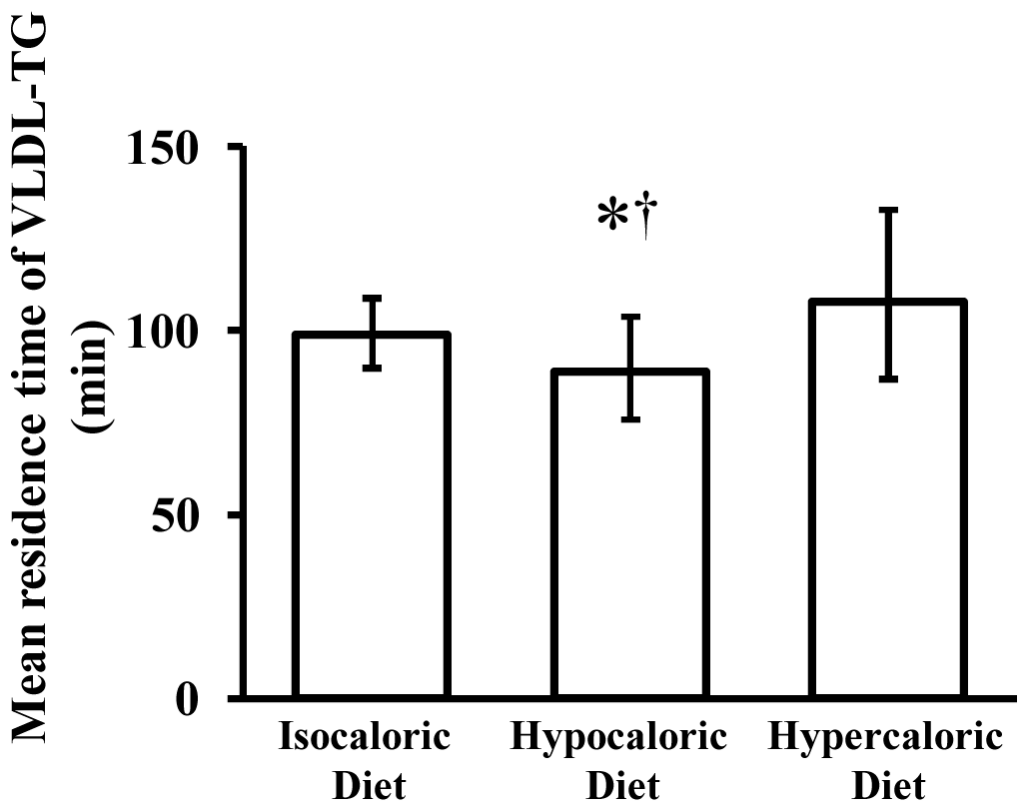
Effects of hypocaloric or hypercaloric feeding relative to isocaloric feeding on the mean residence time of very low density lipoprotein triglyceride (VLDL-TG). Data are means with 95% CI (n = 10). *Significantly different from isocaloric diet, P = 0.044. ^†^ Significantly different from hypercaloric diet, P<0.001.

The fractional turnover rate of VLDL-TG was 0.61±0.08 pools·h^−1^ after isocaloric feeding (control); it increased after the hypocaloric diet (0.69±0.14 pools·h^−1^, P = 0.014 vs. control, P<0.001 vs. overfeeding) but did not change after the hypercaloric diet (0.58±0.17 pools·h^−1^, P = 1.000 vs. control). Compared with the control condition, hepatic VLDL-TG secretion rate was reduced by ∼21% after the hypocaloric diet (P = 0.023) but was not different after the hypercaloric diet (P = 1.000) (Figure 1), whereas the plasma clearance rate of VLDL-TG was increased by ∼12% after the hypocaloric diet (P = 0.016) but was not different after the hypercaloric diet (P = 1.000) (Figure 2). Accordingly, the MRT of VLDL-TG in the circulation was shortened after hypocaloric feeding (P = 0.044) but was not different after hypercaloric feeding (P = 0.850) relative to the control trial (Figure 3).

## Discussion

We evaluated the acute effects of dietary energy restriction (hypocaloric diet) and energy surplus (hypercaloric diet) on basal VLDL-TG kinetics in healthy, lean, sedentary women. Compared with a control day of isocaloric feeding, we found that a single day of dietary energy restriction decreases fasting plasma VLDL-TG concentration by ∼26%, owing to a 21% reduction in hepatic VLDL-TG secretion rate and a 12% increase in the plasma clearance rate of VLDL-TG, whereas dietary energy surplus for a single day has no effects on VLDL-TG concentration and kinetics. The findings from our study indicate that hypotriglyceridemia induced after a single day of dietary restriction in women manifests through a different mechanism (decreased secretion and increased clearance of VLDL-TG) than that described previously after long-term dietary restriction leading to weight loss (decreased secretion of VLDL-TG without changes in VLDL-TG clearance) [3], [5]. The mechanisms underlying the reduction in plasma VLDL-TG concentration after diet-induced energy deficit are therefore the same as those after acute exercise-induced energy deficit in women [8] although at a higher energy cost.

It has been shown previously that long term energy restriction leading to a ∼10% weight loss is accompanied by a 40–50% reduction in hepatic VLDL-TG secretion rate from the liver with no change in VLDL-TG plasma clearance rate in obese women and hypertriglyceridemic men [3], [5]. These effects were apparent after a period of weight stabilization, and were therefore independent of the acute effect of negative energy balance. In another study performed in hypertriglyceridemic subjects, VLDL-TG kinetics were evaluated during active weight loss, and it was shown that 1 month of calorie restriction (energy intake of ∼4 MJ per day) resulted in a marked decrease in the VLDL-TG secretion rate from the liver and a smaller increase in the clearance rate of VLDL-TG from the circulation [28]. Our findings indicate that even a single day of calorie restriction, without the confounding effect of weight loss, also results in a decrease in VLDL-TG secretion rate and an increase in VLDL-TG clearance rate. Collectively, these observations suggest that acute and chronic negative energy balance have the same effects on VLDL-TG metabolism, predominantly decreasing hepatic VLDL-TG secretion and mildly augmenting VLDL-TG clearance. The increase in VLDL-TG plasma clearance rate is not evident when the resulting weight loss is allowed to stabilize (new zero energy balance), implying that the increase in the clearance rate of VLDL-TG is directly linked to negative energy balance.

In a previous study we found that acute calorie restriction of 2 MJ had no effect on basal VLDL-TG concentration and kinetics, although a single bout of moderate intensity aerobic exercise eliciting the same energy deficit lowered fasting plasma VLDL-TG concentrations via both a decrease in VLDL-TG secretion rate and an increase in VLDL-TG clearance rate, in healthy young women [8]. Others have shown that negative energy balance is a critical factor for exercise-induced TG lowering because when dietary energy intake is increased to compensate for the energy expended during exercise, the reduction in fasting total plasma TG concentration is abolished [29]. Our findings suggest that a greater calorie restriction (∼3 MJ) lowers VLDL-TG concentration by the same mechanisms as aerobic exercise in women. However exercise is a more potent stimulus than hypocaloric diet as it requires a lower energy deficit (2 vs 3 MJ) to induce changes in VLDL-TG kinetics leading to hypotriglyceridemia.

In contrast to calorie restriction, the results from the present study indicate that a single day of positive energy balance induced by overfeeding does not have any acute effects on VLDL-TG concentration and metabolism. Our study is the first to evaluate the effects of a single day of hypercaloric feeding with a mixed diet on basal VLDL-TG kinetics. Previous studies reporting changes in VLDL-TG concentration following hypercaloric feeding involved longer periods of overfeeding (4–6 days, usually resulting in mild weight gain) and furthermore examined the effect of hypercaloric feeding with a specific macronutrient (carbohydrate or fat) [9], [10], [11], [12], [13], [14], [15], [30]. These studies have shown that excess fat intake decreases VLDL-TG concentrations [9], [10], [15], whereas excess carbohydrate intake [13], particularly fructose [11], [15], [31], increases VLDL-TG concentrations. Overfeeding with both carbohydrate and fat has no effect on VLDL-TG concentrations [15], [30]. This is in agreement with our results showing that increased caloric intake in a manner mimicking free-living conditions (i.e., overconsumption of all macronutrients) has no effect on basal VLDL-TG metabolism. Nevertheless, we observed that a single day of dietary energy surplus disrupted basal glucose homeostasis. Our finding is in agreement with previous studies showing that 4–5 days of hypercaloric feeding with either fructose [11] or fat [10] result in an increase in fasting plasma glucose concentration [10], [11], and suggests the deleterious effect of overfeeding on glucose homeostasis manifests rapidly. Although this could simply be the result of the greater carbohydrate/glucose load ingested during the hypercaloric diet, the mechanisms for these observations need to be investigated further.

Our findings have several important implications. First, diet-induced negative energy balance can improve TG and VLDL-TG concentrations independently of weight loss; thus people who start on a hypocaloric diet aiming to lose weight will have beneficial effects on TG concentration at a very early stage, even before weight is lost. Second, our findings highlight the importance of weight stabilization (net energy balance) after a period of weight loss in order to evaluate the true effects of weight loss per se on VLDL kinetics [3], [5], because effects attributed to weight loss during the active phase may actually be due to acute negative energy balance. Finally, our results suggest that calorie restriction, like exercise, can acutely improve VLDL-TG concentrations although at a higher energy cost, so both interventions can be utilized to regulate triglyceridemia. An important limitation of the present study is that we only studied healthy lean young women with relatively low plasma TG concentrations, so we cannot generalize our conclusions for men, obese, or hypertriglyceridemic subjects. Nevertheless, hypocaloric feeding decreased VLDL-TG concentrations even further in these subjects, whereas hypercaloric feeding had no effect. Also, we did not evaluate VLDL-apolipoprotein B-100 kinetics in this study, which is indicative of the metabolic behavior of the VLDL particle itself, as opposed to the metabolic behavior of core TG. It has been shown previously that VLDL-TG and VLDL-apoB-100 kinetics are independently regulated, and may be differentially affected by the same intervention, e.g. exercise [32], macronutrient composition of the diet [33], and weight loss [3]. Thus we cannot exclude the possibility that hypocaloric and hypercaloric feeding could affect VLDL-apoB-100 kinetics and thereby lipoprotein metabolism. This possibility needs to be addressed in future studies.

In conclusion, we found that negative energy balance induced by decreasing caloric intake for a single day lowers fasting plasma VLDL-TG concentrations in women via a combination of reduced VLDL-TG secretion from the liver and increased VLDL-TG clearance from plasma. Positive energy balance induced by increasing caloric intake for a single day has no effects on VLDL-TG concentration and kinetics. Collectively, results from the present study indicate that positive and negative energy balance cannot be considered as opposite sides of the same coin in regards to VLDL-TG metabolism.
